# Effects of body weight support training on balance and walking function in stroke patients: a systematic review and meta-analysis

**DOI:** 10.3389/fneur.2024.1413577

**Published:** 2024-08-27

**Authors:** Zhaoxiang Jiang, Xinxin Zhang, Qian Fu, Yimin Tao

**Affiliations:** ^1^College of Physical Education and Health, Guangxi Normal University, Guilin, China; ^2^School of Sports Economics and Management, Guangxi University of Finance and Economics, Nanning, China; ^3^Guilin University of Aerospace Technology, Guilin, China

**Keywords:** body weight support training, stroke, balance, walking function, meta-analysis

## Abstract

**Objective:**

To comprehensively and quantitatively evaluate the impact of body weight support training (BWST) on balance and gait function in stroke patients based on an evidence-based basis and to identify the most effective intervention strategies.

**Methods:**

PubMed, Web of Science, The Cochrane Library, CNKI, Wanfang, and Chinese SinoMed Database were searched until November 25, 2023. Quality assessment and meta-analysis were performed using RevMan 5.2 and Stata 14.0 software.

**Results:**

A total of 31 randomized controlled trials involving 1,918 patients were included in the study. The meta-analysis demonstrated that body weight support training (BWST) significantly improved Berg Balance Scale (BBS) scores (MD = 3.60; 95% CI: 1.23 to 5.98; *p* = 0.003), gait speed (SMD = 0.77; 95% CI: 0.38 to 1.15; *p* < 0.0001), and step length (SMD = 0.46; 95% CI: 0.19 to 0.72; *p* = 0.0008) in stroke patients compared to conventional rehabilitation. For enhancing balance function, the most effective interventions were identified as a disease duration of 3–6 months (MD = 5.16; 95% CI: 0.76 to 9.57; *p* = 0.02), intervention time of 4–8 weeks (MD = 5.70; 95% CI: 2.90 to 8.50; *p* < 0.0001), a maximum body weight support level above 30% (MD = 3.80; 95% CI: 1.48 to 6.13; *p* = 0.001), and a maximum training walking speed of 0.2 m/s or more (MD = 4.66; 95% CI: 0.37 to 9.70; *p* = 0.03). For improving walking function, the optimal interventions were also a disease duration of 3–6 months (gait speed: SMD = 0.59; 95% CI: 0.15 to 1.03; *p* = 0.008; step length: SMD = 0.27; 95% CI: 0.06 to 0.56; *p* = 0.04), intervention time of 4–8 weeks (gait speed: SMD = 1.01; 95% CI: 0.44 to 1.59; *p* = 0.0006; step length: SMD = 0.83; 95% CI: 0.54 to 1.12; *p* < 0.00001), a maximum body weight support level above 30% (gait speed: SMD = 0.79; 95% CI: 0.36 to 1.22; *p* = 0.0003; step length: SMD = 0.79; 95% CI: 0.47 to 1.11; *p* < 0.00001), and a maximum training walking speed of 0.2 m/s or more (gait speed: SMD = 1.26; 95% CI: 0.62 to 1.90; *p* = 0.0001; step length: SMD = 0.85; 95% CI: 0.38 to 1.31; *p* = 0.0003).

**Conclusion:**

Compared with conventional rehabilitation training, BWST demonstrates superior efficacy in enhancing balance and walking function in stroke patients, with a consistent optimal intervention strategy. The most effective program includes a disease duration of 3–6 months, an intervention period of 4–8 weeks, a maximum body weight support of 30% or more, and a maximum training walking speed of 0.2 m/s or greater.

**Systematic review registration:**

http://www.crd.york.ac.uk/PROSPERO/, identifier: CRD42022358963.

## Introduction

Stroke is one of the most prevalent neurological disorders globally. The primary objective of rehabilitation for early-stage stroke patients is to restore lower limb motor function, particularly on balance and walking abilities (1). Previous studies have indicated (1, 2) that over 80% of stroke patients experience balance and walking dysfunction during the acute/subacute phase, characterized by impaired postural alignment, increased sway, asymmetrical gait, and diminished responsiveness to external forces. These impairments significantly reduce patients’ independence and quality of life in performing basic activities of daily living and impose a substantial psychological burden (3, 4). Therefore, finding the best and most effective intervention program for restoring motor function in stroke patients is a key goal for patients (5) and remains a critical scientific issue of significant interest in the field of rehabilitation.

Body weight support training (BWST) is an innovative rehabilitation method that utilizes suspension or pneumatic compression techniques to reduce the effective load of the patient’s body weight during exercise (6). Stroke patients often suffer from overall neurological and motor decline, so their rehabilitation interventions are more demanding than those for single sports injuries (7, 8). BWST is a crucial intervention to reduce weight load, enabling patients to perform comprehensive gait exercises. This approach is better suited to the holistic rehabilitation required by stroke patients than conventional rehabilitation methods and facilitates motor relearning and neural pathway reorganization (9, 10). BWST has been increasingly employed in stroke rehabilitation in recent years, demonstrating notable efficacy (1). A recent study (11) indicated that BWST could significantly enhance lower limb motor function and rehabilitation outcomes, improve patients’ ability to perform daily activities and enhance their quality of life, thereby accelerating their reintegration into family and society. However, previous studies have reported varying results regarding how BWST improves balance and walking function in stroke patients (12, 13). These discrepancies may stem from differences in disease duration and training parameter settings (such as training intensity or intervention time). While earlier research (14–19) has examined individual training parameters (e.g., training time, load), these studies were limited by small sample sizes and the specific characteristics of the included patients, resulting in findings with certain limitations. More importantly, previous intervention trials could only investigate the effect of a single intervention element without exploring the combined effects of different aspects from a multidimensional perspective. Consequently, the optimal intervention program remains undefined.

Therefore, this study aimed to adopt an evidence-based medicine approach to comprehensively and quantitatively assess the effects of BWST on balance and walking function in stroke patients through meta-analysis. Additionally, it sought to identify the optimal intervention program, aiming to discover the most effective rehabilitation strategies for stroke patients and provide valuable references for developing exercise prescriptions.

## Methods

This review was registered (Identifier: CRD42022358963) in the International Prospective Register of Systematic Reviews (PROSPERO) and complied with the Preferred Reporting Items for Systematic Reviews and Meta-Analyses (PRISMA) statement (20).

### Study search and selection

We conducted a comprehensive literature search across PubMed, Web of Science, The Cochrane Library, CNKI, Wanfang, and Chinese SinoMed Databases until November 2023, without any language restrictions. The search terms included (a) Stroke or Cerebral stroke, Cerebral vascular accident or CVA, and (b) Antigravity treadmill or Body weight support. Taking the PubMed database as an example, the specific search strategy is: ((((Stroke [Title/Abstract]) OR (cerebral stroke [Title/Abstract])) OR (cerebral vascular accident [Title/Abstract])) OR (CVA [Title/Abstract])) AND ((antigravity treadmill [Title/Abstract]) OR (body weight support [Title/Abstract])).

The inclusion criteria for this meta-analysis were: (a) the study was a randomized controlled trial, (b) participants were patients with a clinical diagnosis of stroke, (c) interventions involved BWST combined with conventional rehabilitation treatments for the trial group and conventional rehabilitation treatments only for the control group, and (d) the outcomes included Berg Balance Scale (BBS) scores and walking function parameters such as gait speed and step length. The exclusion criteria were: (a) studies that did not involve BWST interventions, (b) interventions that combined BWST with other therapies, (c) patients with other types of diseases, (d) studies with missing or inconsistent outcomes, (e) conference and dissertation papers, and (f) duplicate publications.

EndNote X9 software was used to remove duplicate records from the search results. The title and abstract of retrieved articles were initially read and screened by two reviewers (Z.J., X.Z.) using an independent double-blind approach following the study inclusion and exclusion criteria. Articles that might meet the inclusion criteria were downloaded in full text and read for re-screening to finalize the article’s inclusion. For articles with divergent extractions by two reviewers, a third reviewer (Y.T.) was added to decide on inclusion through joint discussion.

### Data extraction and quality assessment

Two reviewers (Z.J. and X.Z.) independently extracted data from the included articles using a pre-designed form. The extracted information primarily included: (1) basic information about the article, such as the first author and year of publication; (2) basic information about the trial participants, including sample size, age, and disease duration; (3) details of the trial intervention, such as intervention time, frequency, degree of body weight support, and training speed; and (4) baseline and endpoint data of the outcomes.

The risk of bias for the included articles was assessed using the Cochrane Collaboration’s risk-of-bias guidelines (21). This evaluation covered seven key areas: (1) random sequence generation, (2) allocation concealment, (3) blinding of participants and personnel, (4) blinding of outcome assessments, (5) incomplete outcome data, (6) selective reporting, and (7) other biases. Two reviewers (X.Z. and Q.F.) conducted the quality assessment independently. In cases of disagreement, a third reviewer (Y.T.) was involved to reach a consensus through discussion.

### Data synthesis and statistical analysis

Forest plots and subgroup analyses were conducted using RevMan 5.2 software, while funnel plots, sensitivity analyses, meta-regression, and publication bias tests (Egger’s method) were performed using Stata 14.0 software. Effect sizes were reported as mean ± standard deviation with 95% Confidence Intervals (95% CI). Mean Difference (MD) was used for outcomes measured in the same units, while Standardized Mean Difference (SMD) was used for outcomes in different units (22). The Chi^2^ test and I^2^ statistic were used to assess study heterogeneity, with analyses conducted using RevMan 5.2 and Stata 14.0 software. A fixed-effects model was applied if heterogeneity was not statistically significant (I^2^ < 50%; *p* > 0.05); otherwise, a random-effects model was employed (23). Sensitivity analyses were conducted for outcomes with heterogeneity to evaluate the stability of findings, and meta-regression analyses were used to explore sources of heterogeneity. Based on study characteristics, subgroup analyses assessed moderating variables that might influence effect sizes. Statistical significance was set at α = 0.05, with *p* < 0.05 indicating significance.

## Results

### Search results

**Figure 1:**
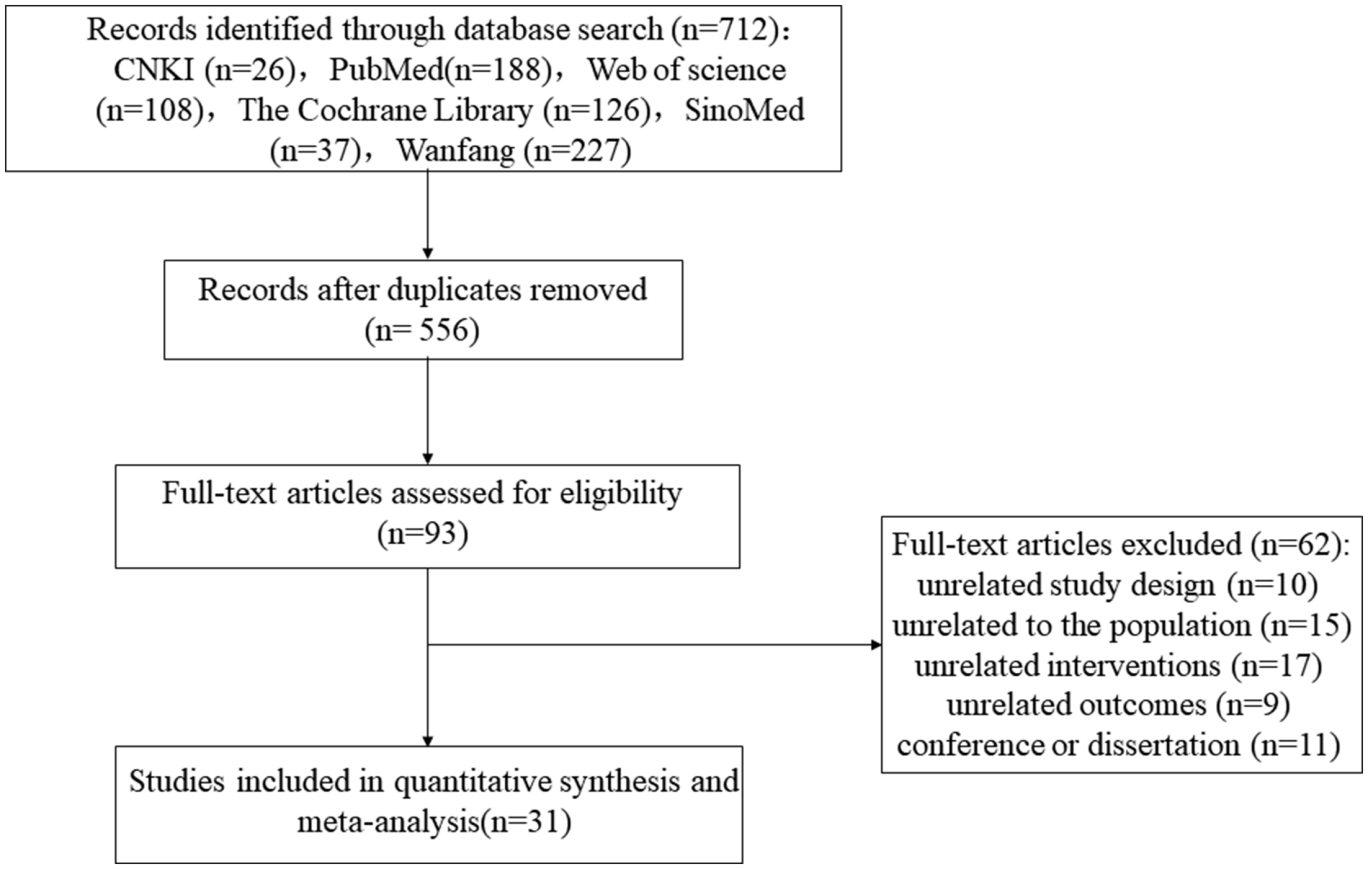
The flow diagram of the selection process.

A total of 712 records were retrieved. After removing duplicates using EndNote, 556 records remained for initial screening. After reviewing titles and abstracts, 463 articles were excluded for irrelevance. The remaining 93 studies were then re-screened through full-text review, excluding 62 studies that did not meet the inclusion criteria. Ultimately, 31 studies were included in the meta-analysis (1, 11–13, 19, 24–49). The literature screening process is depicted in Figure 1.

### Methodological quality assessment

**Figure 2:**
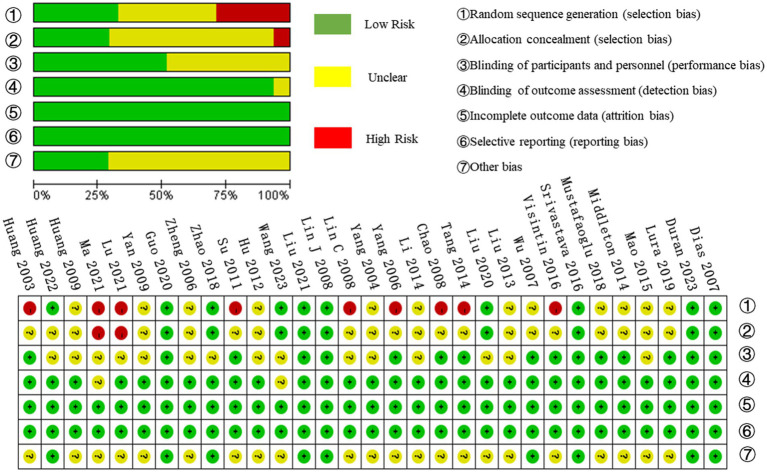
Results of quality evaluation of included studies.

The 31 studies in the meta-analysis involved 1,918 participants, with basic study information detailed in Table 1. The quality of the included studies was assessed using the Cochrane Risk of Bias Assessment Tool. Studies were classified as having a low risk of bias if all seven aspects were evaluated as “low risk.” If one or two aspects were rated as “high risk” or “uncertain risk,” the study was considered to have a moderate risk of bias. Studies with more than two aspects rated as “high risk” or “uncertain risk” were classified as having a high risk of bias. Based on this, six studies were evaluated as low risk of bias (12, 29, 32, 43, 45, 48), four studies were evaluated as moderate risk of bias (11, 19, 42, 44), and 21 studies were evaluated as high risk of bias (1, 13, 24–28, 30, 31, 33–41, 46, 47, 49). All studies had a low risk of incomplete outcome data and selective reporting and a high risk of random sequence generation. The evaluation results are shown in Figure 2.

**Table 1:** The detailed characteristics of each included study.

Year	First author	Sample size (M/F)	Age (Mean ± SD)	Disease duration	Intervention time	Intervention frequency	Maximum body weight support	Training gait speed	Outcomes
2006	Zheng	C: 20/10 E: 25/14	51.2	20–130 days	8–16 weeks	10-15 min/times, 1 time/day, 6 days/week	30–60%	0.25 m/s	Gait speed
2018	Zhao	C: 12/6 E: 11/7	C: 63.7 ± 9.6 E: 65.3 ± 8.1	C: 6.31 ± 2.47 months E: 5.92 ± 2.13 months	4 weeks	30 min/times, 2 time/day, 5 days/week	30–40%	0.2–0.4 m/s	BBS Score
2006	Yang	35/23	53.21 ± 9.68	NR	8 weeks	15-30 min/times, 1 time/ day, 5 days/week	30–40%	NR	Gait speed, step length
2004	Zhao	C: 21/9 E: 15/6	C: 55.15 ± 10.71 E: 54.00 ± 10.71	C: 127.58 ± 68.33 days E: 130.25 ± 64.53 days	6 weeks	30 min/times, 1 time/day, 5 days/week	30–40%	0.14 m/s	Gait speed, step length
2009	Yan	C: 17/8 E: 19/6	C: 55.2 ± 10.9 E: 57.6 ± 10.6	C: 78.8 ± 40.3 days E: 80.6 ± 38.5 days	6 weeks	5-20 min/times, 1 time/ day, 6 days/week	40–50%	10 cm/s	BBS Score
2007	Wu	C: 13/7 0% E: 14/6 30% E: 13/7	C: 57.6 ± 10.8 0%E: 57.3 ± 12.5 30%E: 58.2 ± 11.6	C: 113.8 ± 45.0 days 0%E: 110.8 ± 46.5 days 30%E: 116.2 ± 42.3 days	4 weeks	30 min/times, 1 time/ day, 5 days/week	0或30%	0% E:0.1 m/s 30% E:0.15 m/s	BBS Score, Gait speed, step length
2023	Wang	E: 7/21\u00B0C: 16/16	C: 58.63 ± 5.53 E: 58.81 ± 5.39	C: 22.43 ± 3.08 days E: 22.78 ± 3.25 days	2 months	30 min/times, 1 time/day, 5 days/week	NR	0.27 m/s	BBS Score, Gait speed, step length
2014	Tang	C: 20 E: 20	NR	NR	4 weeks	20-30 min/times, 1 time/ day, 5 days/week	30%	0.01 m/s	BBS Score
2011	Su	43/29	62.8 ± 7.3	NR	NR	NR	NR	NR	BBS Score
2021	Ma	C: 37/23 E: 39/21	C: 58.43 ± 2.14 E: 58.40 ± 2.11	C: 19.78 ± 1.63 days E: 19.81 ± 1.65 days	4 weeks	20-30 min/times, 1 time/day	NR	NR	BBS Score
2021	Lu	C: 28/20 E: 30/18	C: 62.89 ± 3.47 E: 63.02 ± 3.51	NR	28 days	20-30 min/times, 3 time/week	NR	NR	BBS Score
2021	Liu	C: 25/15 E: 26/14	C: 52.1 ± 13.4 E: 53.1 ± 12.8	C: 12.7 ± 4.2 days E: 11.2 ± 4.6 days	4 weeks	30 min/times, 1 time/day, 6 days/week	35%	0.15–0.45 m/s	BBS Score
2013	Liu	C: 17/7 E: 18/6	C: 56.08 ± 5.99 E: 55.23 ± 7.06	C: 60.67 ± 5.95 days E: 61.38 ± 7.56 days	12 weeks	15 min/times, 2 time/day, 5 days/week	NR	NR	BBS Score
2020	Liu	C: 25/22 E: 27/20	C: 58.67 ± 4.12 E: 59.02 ± 4.56	C: 3.51 ± 1.52 months E: 3.67 ± 1.42 months	8 weeks	30 min/times, 1 time/day	NR	NR	BBS Score, Gait speed
2008	Lin C	C: 12/8 E: 11/9	C: 65.5 ± 7.3 E: 66.2 ± 2.4	C: 35–60 days E: 32–58 days	8 weeks	5-30 min/times, 2 time/day, 5 days/week	NR	NR	BBS Score
2008	Lin J	C: 16/7 E: 15/8	C: 53.6 ± 10.2 E: 51.3 ± 10.8	C: 28.7 ± 16.7 days E: 30.5 ± 15.3 days	4–6 weeks	10-30 min/times, 1 time/ day, 5 days/week	40%	0.2-2 m/s	BBS Score
2014	Li	C: 56/44 E: 58/42	C: 61.3 ± 8.9 E: 59.4 ± 9.7	C: 18.1 ± 9.0 days E: 16.4 ± 9.3 days	4 weeks	15-20 min/times, 1 time/day, 5 days/week	40%	0.2–0.4 m/s	BBS Score
2009	Huang	C: 20/11 E: 18/14	C: 58.3 ± 13.4 E: 60.5 ± 11.3	C: 16.5 ± 9.7 days E: 15.3 ± 10.4 days	6 weeks	15-20 min/times, 1 time/day	30%	0.5 m/s	BBS Score
2003	Huang	C: 5/7 E: 5/7	C: 58.3 ± 9.65 E: 57.5 ± 10.56	C: 20.58 ± 14.30 days E: 22.08 ± 25.31 days	2 weeks	5-30 min/times, 1 time/day, 5 days/week	30%	0.2 km/h	BBS Score, Gait speed
2022	Huang	C: 28/22 E: 31/19	C: 53.13 ± 8.23 E: 49.27 ± 5.17	C: 2.48 ± 1.68 years E: 2.74 ± 1.52 years	12 weeks	More than 20 min /times, 1 time/day, 5 days/week	30%	0.15 m/s	Gait speed, step length
2012	Hu	C: 21/13 E: 19/12	C: 61.4 ± 8.5 E: 62.8 ± 7.3	NR	8 weeks	15-30 min/times, 1 time/day, 5 days/week	30–40%	NR	step length, Gait speed
2020	Guo	C: 17/13 E: 15/15	C: 58.4 ± 7.5 E: 57.9 ± 6.4	C: 3.95 ± 1.34 months E: 3.76 ± 1.14 months	2 weeks	30 min/times, 1 time/day, 5 days/week	NR	NR	Gait speed, step length
2008	Chao	37/23	66.7 ± 3.8	NR	8 weeks	15-30 min/times, 1 time/day, 5 days/week	30–40%	NR	BBS Score
1998	Visintin	C: 31/19 E: 30/20	C: 66.7 ± 10.1 E: 66.5 ± 12.8	C: 78.4 ± 30.0 days E: 68.1 ± 26.5 days	6 weeks	Less than 20 min /times, 4 time/week	40%	0–0.1 m/h	BBS Score, Gait speed
2016	Srivastava	C: 12/3 E: 12/3	C: 47.93 ± 9.95 E: 44.2 ± 11.7	C: 442.07 ± 295.13 days E: 391.80 ± 431.10 days	4 weeks	20 min/times, 5 time/week	40%	0–0.16 km/h	Gait speed
2018	Mustafaoglu	C: 11/4 E: 11/4	C: 52.6 ± 14.7 E: 53.7 ± 11.6	C: 11 months E: 12 months	6 weeks	45 min/times, 2 time/week	30–40%	1.2–2.6 km/h	BBS Score
2014	Middleton	C: 16/4 E: 14/9	C: 60.70 ± 14.43 E: 61.39 ± 15.69	C: 29.03 ± 23.90 months E: 50.41 ± 56.80 months	10 weeks	NR	8–50%	NR	BBS Score, step length
2015	Mao	C: 2/7 E: 2/8	C: 60.82 ± 10.7 E: 59.55 ± 9.23	C: 47.67 ± 16.78 days E: 49.25 ± 19.51 days	3 weeks	NR	30–40%	0.5–2.5 m/s	Gait speed, step length
2019	Lura	C: 12/8 E: 15/3	C: 60.4 ± 16.1 E: 63.8 ± 10.8	C: 18.1 ± 4.1 days E: 23.5 ± 8.9 days	NR	NR	NR	NR	step length, Gait speed
2023	Duran	C: 13 E: 13	C: 57.9 ± 10.9 E: 54.1 ± 18.9	C: 10.0 ± 5.1 months E: 12.0 ± 4.0 months	4 weeks	12 times/day, 3 days/week	65–100%	0-2 m/h	BBS Score
2007	Dias	C: 14/6 E: 16/4	C: 68.0 ± 10.69 E: 70.35 ± 7.36	C: 48.45 ± 29.51 days E: 47.10 ± 63.83 days	5 weeks	40 min/times, 5 time/week	30%	NR	BBS Score

E, experimental group; C, control group; NR, not reported; BBS Score, Berg Balance Scale Score.

### Meta-analytic results

#### Effect of BWST on BBS scores

**Figure 3:**
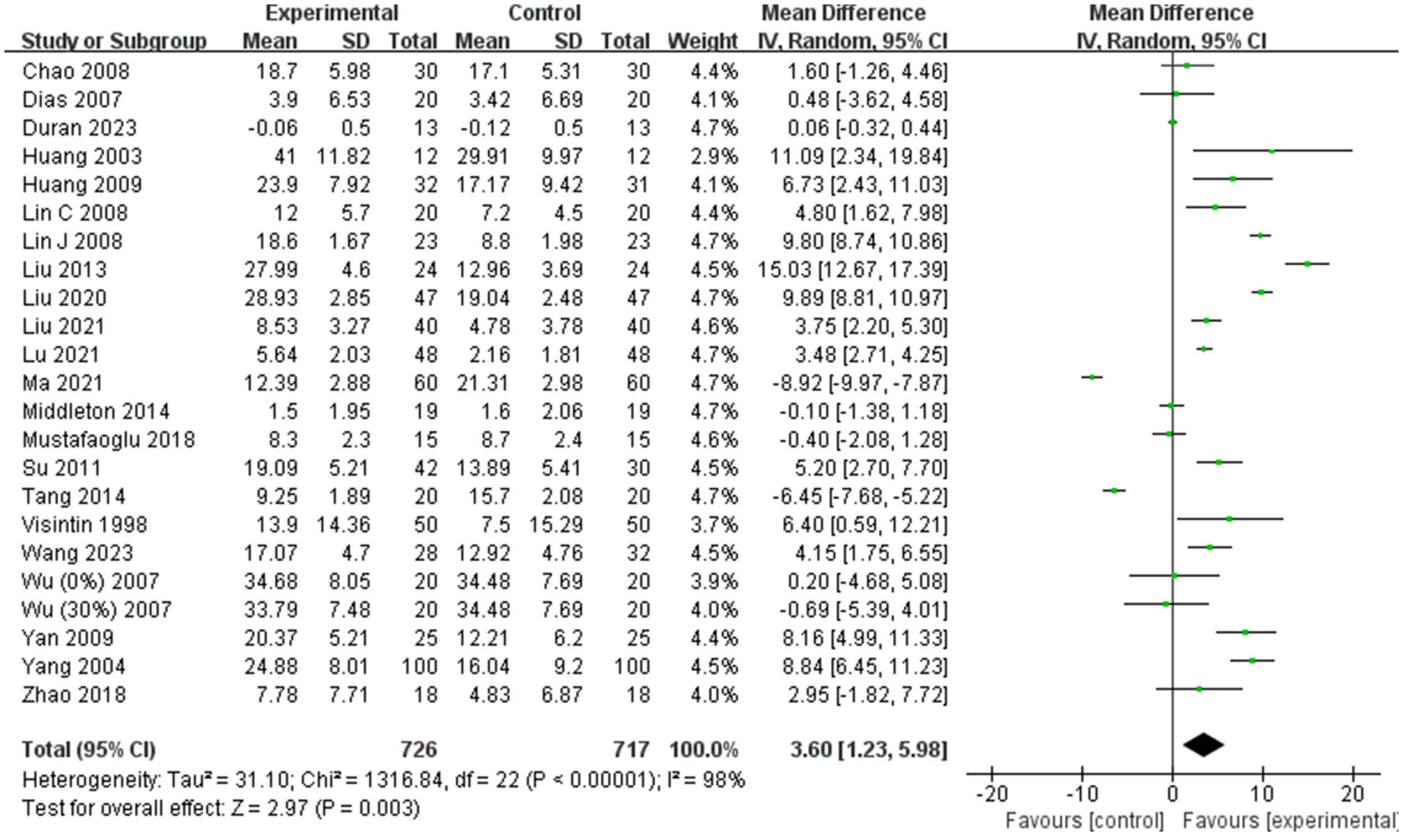
Effects of BWST on BBS scores.

Twenty-two of the 31 included studies (1, 19, 24–26, 29–35, 37, 38, 40, 42, 44–49) were analyzed for BBS scores. Due to high heterogeneity among the combined results (I^2^ = 98%), a random effects model was employed for the meta-analysis. The combined effect size indicated a significant improvement in BBS scores in the BWST group compared to the control group (MD = 3.60; 95% CI: 1.23–5.98; *p* = 0.003). The results are presented in Figure 3.

To investigate the effects of relevant moderating variables on the results of the study, four moderating variables were extracted based on the characteristics of the included studies, namely, patients’ disease duration, intervention time, maximum degree of body weight support, and maximum training gait speed. A subgroup analysis based on the above moderating variables found (as shown in Table 2) that the intervention was most effective for patients with a disease duration of 3–6 months (MD = 5.16; 95% CI: 0.76 to 9.57; *p* = 0.02). No statistically significant effects were observed for patients with a disease duration of 1–3 months (MD = 4.78; 95% CI: −1.80 to 11.36; *p* = 0.15) or more than 6 months (MD = 0.04; 95% CI: −0.32 to 0.40; *p* = 0.82). The most effective treatment was observed with an intervention duration of 4–8 weeks (MD = 5.70; 95% CI: 2.90–8.50; *p* < 0.0001). Interventions lasting 1–4 weeks (MD = −0.04; 95% CI: −3.28 to 3.19; *p* = 0.98) or more than 8 weeks (MD = 6.32; 95% CI: −2.61 to 15.26; *p* = 0.17) showed no statistically significant effect. An intervention with a maximum body weight support of 30% or more was effective (MD = 3.80; 95% CI: 1.48–6.13; *p* = 0.001). Support of 0–30% did not show a statistically significant treatment effect (MD = 1.34; 95% CI: −4.04 to 6.73; *p* = 0.62). A maximum training gait speed of 0.2 m/s or more significantly improved balance function (MD = 4.66; 95% CI: 0.37–9.70; *p* = 0.03), while speeds of 0–0.2 m/s did not show a statistically significant effect (MD = 2.96; 95% CI: −0.83 to 6.75; *p* = 0.13). In conclusion, BWST was most effective in improving BBS scores in stroke patients with a disease duration of 3–6 months, an intervention time of 4–8 weeks, a maximum body weight support of 30% or more, and a maximum training gait speed of 0.2 m/s or more.

**Table 2:** Subgroup analysis of moderating variables affecting BBS scores.

Moderating variables	Subgroup	Sample size	Number of studies	Effect size and 95% CI	I^2^ (%)	*p*-value
Disease duration	1–3 months	483	11	4.78 (−1.80, 11.36)	99	0.15
	3–6 months	374	4	5.16 (0.76, 9.57)	90	0.02
	More than 6 months	130	4	0.04 (−0.32, 0.40)	0	0.82
Intervention time	1–4 weeks	502	9	−0.04 (−3.28, 3.19)	98	0.98
	4–8 weeks	723	10	5.70 (2.90, 8.50)	94	<0.0001
	More than 8 weeks	146	3	6.32 (−2.61, 15.26)	98	0.17
Maximum degree of body weight support	0–30%	247	6	1.34 (−4.04, 6.73)	92	0.62
	More than 30%	622	9	3.80 (1.48, 6.13)	92	0.001
Maximum training gait speed	0–0.2 m/s	520	8	2.96 (−0.83, 6.75)	96	0.13
	More than 0.2 m/s	235	5	4.66 (0.37, 9.70)	96	0.03

Additionally, meta-regression analysis was conducted to identify significant factors influencing heterogeneity when the number of studies exceeded ten and the heterogeneity I^2^ was >50%. The results (shown in Table 3) indicated that intervention time (*p* = 0.319), maximum body weight support (*p* = 0.302), and maximum training gait speed (*p* = 0.441) did not significantly contribute to heterogeneity. In contrast, disease duration showed a statistically significant result (*p* = 0.046), suggesting it may be the primary source of heterogeneity.

**Table 3:** Meta-regression analysis of different moderating variables on BBS scores.

Moderating variables	β-regression coefficient	Standard error	*t*-value	P>│*t*│	95%CI
Disease duration	1.600385	0.9079024	1.76	0.046	(0.3151214, 3.515892)
Intervention time	−1.030576	1.009444	−1.02	0.319	(−3.13624, 1.075088)
Maximum degree of body weight support	−1.481964	1.3782	−1.08	0.302	(−4.459383, 1.495456)
Maximum training gait speed	−1.152835	1.44286	−0.8	0.441	(−4.328549, 2.022878)

#### Effect of BWST on walking function

**Figure 4:**
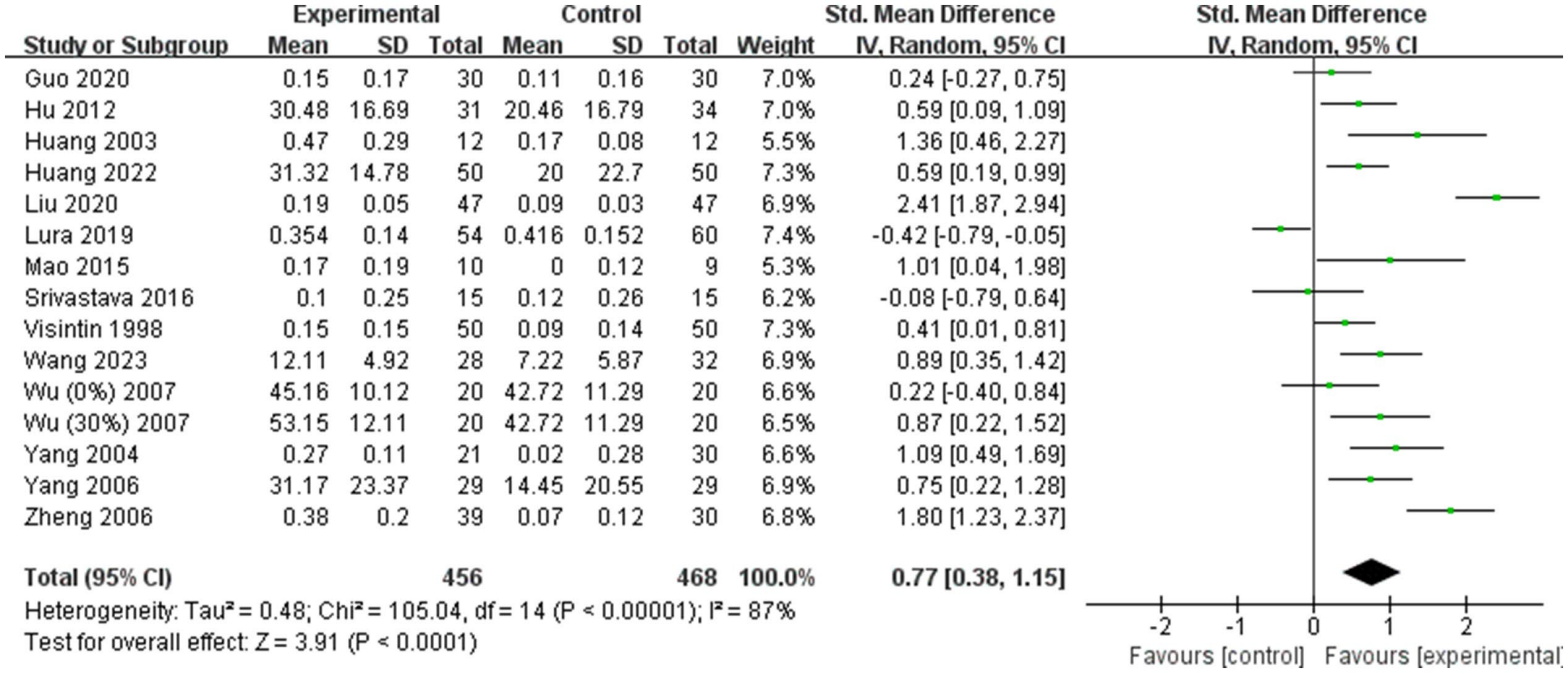
Effect of BWST on gait speed.

**Figure 5:**
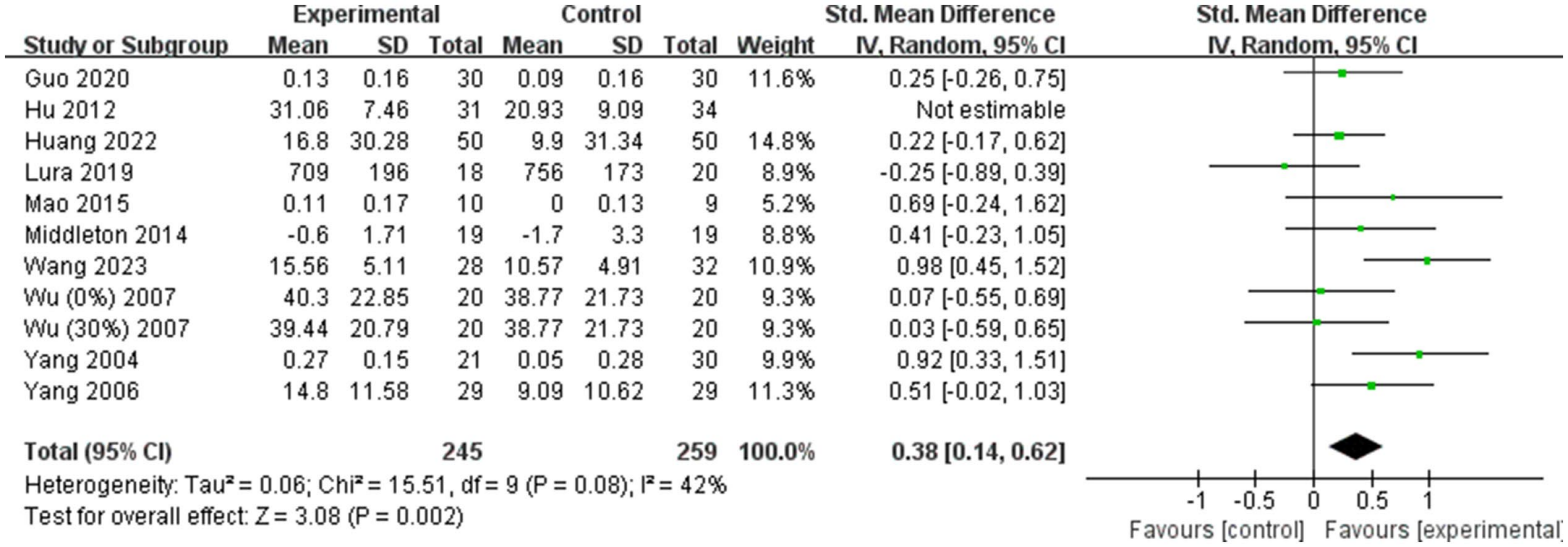
Effect of BWST on step length.

A total of 14 (11–13, 19, 24–28, 36, 41, 43, 44, 49) of the 31 included studies analyzed gait speed. Due to significant heterogeneity among the results (I^2^ = 87%), a random effects model was employed for the meta-analysis. The combined effect size demonstrated a significantly greater gait speed in the BWST group than in the control group (SMD = 0.77; 95% CI: 0.38 to 1.15; *p* < 0.0001, Figure 4). Additionally, 10 (11, 13, 19, 26, 27, 36, 38, 41, 43, 49) of the 31 studies included analyzed step length. Given the large heterogeneity in the combined results (I^2^ = 58%), a random effects model was also used for the meta-analysis. The combined effect size revealed a significantly greater improvement in step length for the BWST group compared to the control group (SMD = 0.46; 95% CI: 0.19–0.72; *p* = 0.0008, Figure 5).

Also, subgroup analyses were conducted to explore the impact of relevant moderating variables—disease duration, intervention time, maximum degree of body weight support, and maximum training gait speed—on study outcomes (Table 4). It was found that in terms of the gait speed outcome, the intervention was most effective for patients with a disease duration of 3–6 months (SMD = 0.59; 95% CI: 0.15–1.03; *p* = 0.008). No significant effects were observed for durations of 1–3 months (SMD = 0.57; 95% CI: −0.07 to 1.22; *p* = 0.08) or more than 6 months (SMD = 0.32; 95% CI: −0.31 to 0.96; *p* = 0.32). An intervention time of 4–8 weeks showed superior results (SMD = 1.01; 95% CI: 0.44–1.59; *p* = 0.0006) compared to 1–4 weeks (SMD = 0.53; 95% CI: 0.12–0.93; *p* = 0.01). An intervention duration of more than 8 weeks (SMD = 1.18; 95% CI: −0.01 to 2.37; *p* = 0.05) was not statistically significant. Maximum body weight support of 30% or more was more effective (SMD = 0.79; 95% CI: 0.36–1.22; *p* = 0.0003) than support of 0–30% (SMD = 0.67; 95% CI: 0.29–1.05; *p* < 0.00001). A maximum gait speed of 0.2 m/s or more resulted in better outcomes (SMD = 1.26; 95% CI: 0.62–1.90; *p* = 0.0001) compared to speeds of 0–0.2 m/s (SMD = 0.60; 95% CI: 0.29–0.90; p < 0.00001). For the step length outcome, the intervention was most effective for a disease duration of 3–6 months (SMD = 0.27; 95% CI: 0.06–0.56; *p* = 0.04). No significant effects were observed for durations of 1–3 months (SMD = 0.44; 95% CI: −0.33 to 1.21; *p* = 0.26) or more than 6 months (SMD = 0.30; 95% CI: −0.03 to 0.64; *p* = 0.08). The best results were achieved with an intervention time of 4–8 weeks (SMD = 0.83; 95% CI: 0.54–1.12; *p* < 0.00001). Interventions lasting 1–4 weeks (SMD = 0.17; 95% CI: −0.15 to 0.48; *p* = 0.29) or more than 8 weeks (SMD = 0.30; 95% CI: −0.03 to 0.64; p = 0.08) showed no statistically significant effects. A maximum body weight support of 30% or more was most effective (SMD = 0.79; 95% CI: 0.47–1.11; p < 0.00001). Support of 0–30% (SMD = 0.14; 95% CI: −0.15 to 0.43; *p* = 0.35) was not statistically significant. A maximum gait speed of 0.2 m/s or more yielded the best results (SMD = 0.85; 95% CI: 0.38–1.31; *p* = 0.0003), while speeds of 0–0.2 m/s (SMD = 0.26; 95% CI: −0.02 to 0.54; *p* = 0.05) showed no significant effect. In summary, BWST with a disease duration of 3–6 months, an intervention time of 4–8 weeks, a maximum body weight support of 30% or more, and a maximum training gait speed of 0.2 m/s or more demonstrated the most effective improvement in balance and walking function in stroke patients.

**Table 4:** Subgroup analysis of moderating variables affecting walking function.

Outcomes	Moderating variables	Subgroup	Sample size	Number of studies	Effect size and 95% CI	I^2^ (%)	P-value
Gait speed	Disease duration	1–3 months	317	5	0.57 (−0.07, 1.22)	85	0.08
		3–6 months	191	4	0.59 (0.15, 1.03)	54	0.008
		More than 6 months	130	2	0.32 (−0.31, 0.96)	60	0.32
	Intervention time	1–4 weeks	213	6	0.53 (0.12, 0.93)	50	0.01
		4–8 weeks	428	6	1.01 (0.44, 1.59)	87	0.0006
		More than 8 weeks	169	2	1.18 (−0.01, 2.37)	91	0.05
	Maximum degree of body weight support	0–30%	204	4	0.67 (0.29, 1.05)	37	<0.00001
		More than 30%	392	7	0.79 (0.36, 1.22)	74	0.0003
	Maximum training gait speed	0–0.2 m/s	385	7	0.60 (0.29, 0.90)	48	<0.00001
		More than 0.2 m/s	148	3	1.26 (0.62, 1.90)	65	0.0001
Step length	Disease duration	1–3 months	117	3	0.44 (−0.33, 1.21)	74	0.26
		3–6 months	191	4	0.27 (0.06, 0.56)	12	0.04
		More than 6 months	138	2	0.30 (−0.03, 0.64)	0	0.08
	Intervention time	1–4 weeks	159	4	0.17 (−0.15, 0.48)	0	0.29
		4–8 weeks	234	4	0.83 (0.54, 1.12)	14	<0.00001
		More than 8 weeks	138	2	0.30 (−0.03, 0.64)	0	0.08
	Maximum degree of body weight support	0–30%	180	3	0.14 (−0.15, 0.43)	0	0.35
		More than 30%	193	4	0.79 (0.47, 1.11)	13	<0.00001
	Maximum training gait speed	0–0.2 m/s	231	4	0.26 (−0.02, 0.54)	11	0.05
		More than 0.2 m/s	79	2	0.85 (0.38, 1.31)	0	0.0003

Meta-regression analysis showed (as shown in Table 5) that the test results for heterogeneity of the disease duration, intervention time, maximum body weight support and maximum training gait speed were not statistically different.

**Table 5:** Meta-regression analysis of the effects of different moderating variables on walking function.

Outcomes	Moderating variables	β-regression coefficient	Standard error	*t*-value	P>│*t*│	95%CI
Gait speed	Disease duration	0.6946287	0.4423174	1.57	0.151	(−0.3059628, 1.69522)
	Intervention time	0.2912388	0.4771275	0.61	0.553	(−0.7483327, 1.33081)
	Maximum degree of body weight support	0.6472511	0.6014062	1.08	0.31	(−0.7132243, 2.007727)
	Maximum training gait speed	−0.0573704	0.4608746	−0.12	0.904	(−1.120149, 1.005408)
Step length	Disease duration	0.5537579	0.4223577	1.31	0.231	(−0.4449593, 1.552475)
	Intervention time	0.3312973	0.379584	0.87	0.408	(−0.5440249, 1.20662)
	Maximum degree of body weight support	−0.5708826	0.335209	−1.7	0.149	(−1.432565, 0.2907995)
	Maximum training gait speed	−0.2896365	0.4585486	−0.63	0.562	(−1.562771, 0.9834984)

### Sensitivity analyses

**Figure 6:**
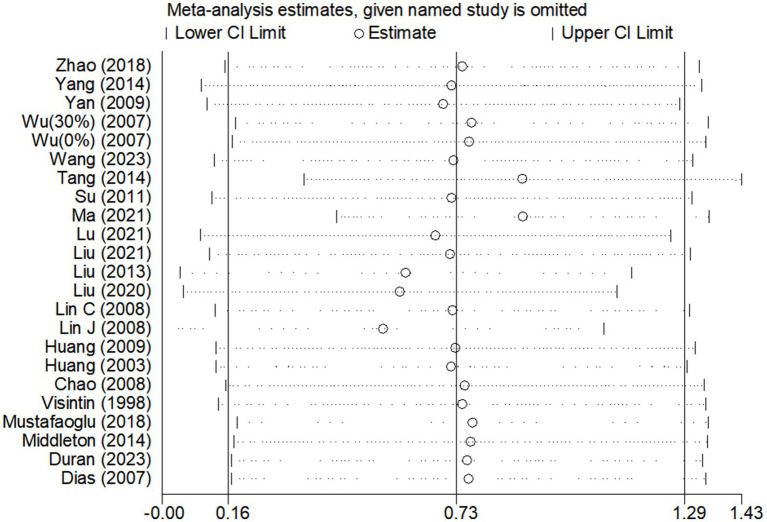
Sensitivity analysis of BWST on BBS scores.

**Figure 7:**
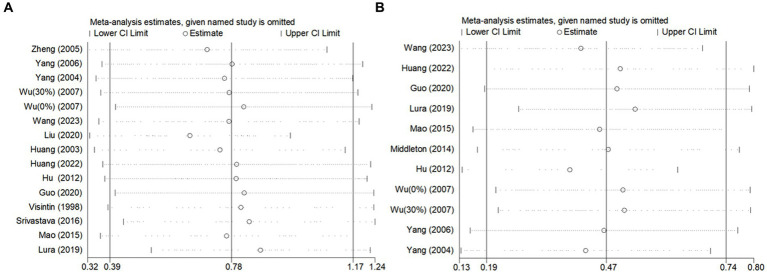
Sensitivity analysis of the effect of BWST on walking function (**A**: gait speed, **B**: step length).

Due to the high degree of heterogeneity between studies, a study-by-study culling approach was adopted to assess the impact of a single study on the overall effect size based on the overall study. The analysis revealed that the exclusion of single studies had minimal impact on the overall effect sizes for BBS scores (Figure 6) and gait speed (Figure 7A), indicating the robustness and reliability of the findings for these outcomes.

However, for step length (Figure 7B), the heterogeneity was significantly reduced (I^2^ = 42%) following the exclusion of the study by Hu (36). This suggests that Hu’s study may have been a significant source of heterogeneity in the step length outcome. Despite this reduction in heterogeneity, the overall effect size for step length remained consistent before and after the exclusion of Hu’s study (Post-exclusion: SMD = 0.38; 95% CI: 0.14 to 0.62; *p* = 0.002). This stability in the effect size confirms that the findings regarding step length are reliable and not unduly influenced by a single study.

### Publication bias

**Figure 8:**
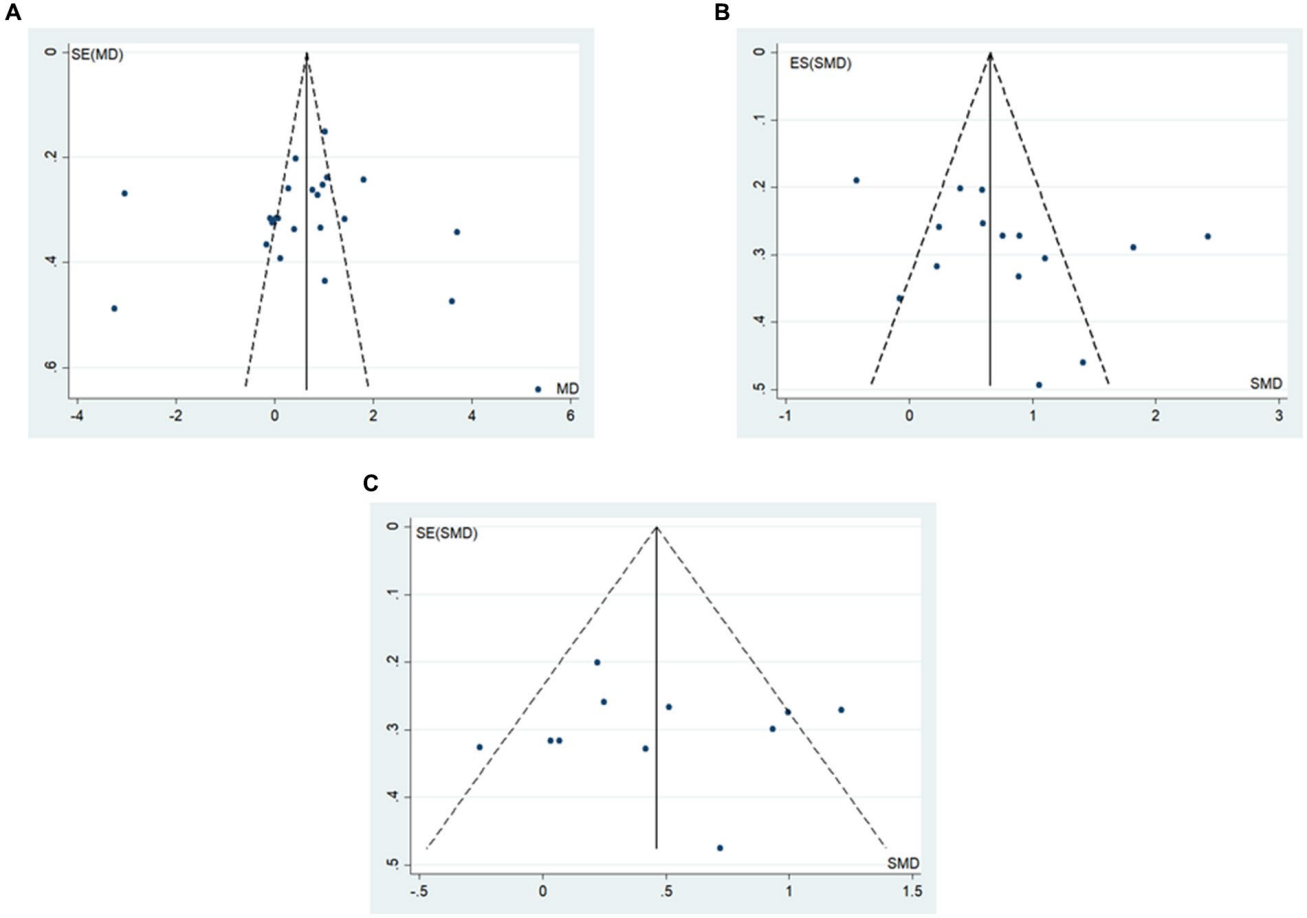
Meta-analysis publication bias funnel plot (**A**: BBS score, **B**: gait speed, **C**: step length).

The funnel plot and Egger’s method were utilized to assess publication bias. The results indicated that BBS scores and walking function outcomes were symmetrically distributed around the funnel plot (Figure 8). Additionally, the Egger’s test did not reveal statistically significant differences (*p* > 0.05) (Table 6), suggesting a low probability of publication bias in the study’s outcomes.

**Table 6:** Egger’s method test results.

Outcomes	Std_Eff	Coef.	Standard error	*t*-value	P>│*t*│	95%CI
BBS scores	Slope	0.4791136	0.9686618	0.49	0.626	(−1.535329, 2.493556)
	Bias	0.6317084	3.388125	0.19	0.854	(−6.414283, 7.6777)
Gait speed	Slope	−0.4889524	0.7478258	−0.65	0.525	(−2.104532, 1.126627)
	Bias	4.369748	2.764352	1.58	0.138	(−1.602271, 10.34177)
Step length	Slope	0.3983997	0.7386791	0.54	0.603	(−1.272608, 2.069408)
	Bias	0.2267712	2.586182	0.09	0.932	(−5.62358, 6.077122)

## Discussion

Balance and walking deficits resulting from lower limb motor dysfunction are critical factors influencing stroke patients’ ability to return to self-care and reintegrate into family and society (11). The meta-analysis conducted in this study demonstrated that BWST significantly enhances balance and walking function in stroke patients compared to conventional rehabilitation training, aligning with findings from previous research (12, 13).

Several previous studies have confirmed the validity of BBS scores, gait speed and step length as effective measures for evaluating balance and walking function in patients (12, 13). Additionally, the theoretical foundation of BWST in improving motor function is well-established (9, 10), drawing from central pattern generator theory, motor control dynamical system theory, and the theory of compulsory use (9). The mechanism of BWST involves mandatory exercise with reduced body weight and regulated gait speed, which enhances leg coordination in stroke patients (25) and improves motor relearning and neural pathway reorganization (9, 10).

During training, the therapist can integrate weight bearing, stepping, and balance components by adjusting training loads and body weight support according to the patient’s pathology, thereby enhancing proprioceptive input to the lumbar spinal cord and optimizing motor neural pathways, which promotes the consolidation of normal motor patterns (28). Moreover, from a psychological point of view, walking training with adequate safety measures provides patients with a sense of security, reducing anxiety and fear of falling (28). In summary, previous studies have addressed the mechanism of BWST to improve patients’ motor function in terms of movement pattern control and development, neural pathway conduction, and psychology. While the mechanisms underlying BWST’s effects on motor function are well-explored, there is limited research on the impact of individual differences, training intensity, and duration. Future studies should investigate these aspects further to provide a comprehensive understanding of BWST’s therapeutic effects.

The impact of BWST timing parameters on intervention outcomes can be examined through two key dimensions: the initiation and duration of the intervention. This study’s subgroup analysis revealed that the intervention time of 4–8 weeks was the best intervention program to improve stroke patients’ balance and walking function during 3–6 months of the patient’s disease duration. Hayes et al. (50) noted that early rehabilitation training can facilitate faster recovery of lower limb function in post-stroke patients, promoting brain cell regeneration around lesions and enhancing motor function compensation and reorganization in the contralateral cerebral hemisphere (51). However, the specific timing of BWST relative to routine rehabilitation is unclear. Song et al. (52) found that BWST is appropriate for patients with stable conditions and lower limb muscle strength of grade 3 or higher. Tong et al. (18) underscored the efficacy of early BWST intervention for recovering lower limb walking function in stroke patients, particularly within the first month of disease duration. Nevertheless, their study focused on patients with a disease duration of less than two months, limiting insights into longer durations. In addition, different intervention times also have a greater impact on the treatment effects of patients, but there are few comparisons of treatment effects based on different intervention cycles. This study demonstrates that for stroke patients with a disease duration of 3–6 months, an intervention period of 4–8 weeks yields the most favorable outcomes. Further research should explore these findings by including diverse patient populations and controlling intervention timing through randomized controlled trials.

The impact of training load parameters on intervention outcomes is evident in two primary areas: the degree of body weight support and the training gait speed. Previous studies have yielded varying results regarding the optimal degree of body weight support. Liu et al. (15) found that exceeding 50% of the maximum body weight support could induce gait abnormalities, hindering motor function recovery. Conversely, Hesse et al. (53) recommended that body weight support should not surpass 30%. However, the present study’s subgroup analysis demonstrated that a maximum body weight support exceeding 30% was more effective for rehabilitation, which diverges from earlier findings. This discrepancy may be attributed to differences in patient characteristics, as previous studies (15, 53) had small sample sizes and patients with a disease duration of approximately 40 days, potentially limiting their conclusions. Expanding patient characteristics and sample size in this study may account for the differing results, warranting further investigation.

Regarding setting the maximum training gait speed, Van et al. (54) argued against using lower speeds for gait training, as it might diminish muscle activation and cause abnormal gait patterns. Wu Hua et al. (16) found that a maximum gait training speed of 0.3 m/s yielded the most significant motor feedback improvement in stroke patients, whereas higher speeds (0.45 m/s) did not enhance motor function. The current study indicates that a maximum training gait speed of 0.2 m/s or more provides the most effective therapeutic outcomes for balance and walking function in stroke patients, suggesting that lower speeds are less effective, though the impact of higher speeds remains unclear. Proper adjustment of training loads according to patient mobility is crucial (55). For less mobile patients, increased body weight support or lower gait speeds may facilitate recovery, while more mobile patients might benefit less. This study’s subgroup analyses did not account for variations in patient mobility, and the limitations of included studies restricted further refinement of body weight support and gait speed classifications. Future research should explore the effects of varying body weight support ratios and gait speeds on recovery outcomes by integrating patient characteristics and training loads.

Despite the comprehensive analysis and assessment of all eligible studies, this review has several limitations. Firstly, many of the included studies were of low quality and had a certain risk of bias, which may affect the reliability of the study conclusions. Secondly, literature was excluded due to the absence of relevant outcomes, suggesting that the range of outcomes could be expanded in future research. Thirdly, some subgroups in the analyses were based on a limited number of studies, and the objectivity of these conclusions needs further validation. Future research should focus on randomized controlled trials with larger sample sizes to enhance the robustness of the findings. Additionally, further discussions should explore the impact of variations in patient characteristics and interventions to validate the conclusions of this study.

## Conclusion

Compared to conventional rehabilitation, BWST demonstrated superior effectiveness in enhancing balance and walking function in stroke patients. The optimal intervention protocol identified was a 4–8 week treatment time, with a maximum body weight support of 30% or more, and a maximum training gait speed of 0.2 m/s or higher, applied during a 3–6 months disease duration.

## Data Availability

The original contributions presented in the study are included in the article/supplementary material, further inquiries can be directed to the corresponding author.
